# Thyromental height test as a new method for prediction of difficult intubation with double lumen tube

**DOI:** 10.1371/journal.pone.0201944

**Published:** 2018-09-13

**Authors:** Piotr Palczynski, Szymon Bialka, Hanna Misiolek, Maja Copik, Anna Smelik, Lukasz Szarpak, Kurt Ruetzler

**Affiliations:** 1 Department of Anaesthesiology and Intensive Care, Medical University of Silesia, Katowice, Poland; 2 Lazarski University, Warsaw, Poland; 3 Departments of Outcomes Research and General Anesthesiology, Anesthesiology Institute, Cleveland Clinic, Cleveland, Ohio, United States of America; Imam Abdulrahman Bin Faisal University College of Medicine, SAUDI ARABIA

## Abstract

**Background:**

Predicting difficult intubation is of high clinical interest.

**Methods:**

237 patients aged ≥18 years were included in the study. Preoperative airway evaluation included: Mallampati test, thyromental distance, sternomental distance and thyromental height test. During direct laryngoscopy Cormack & Lehane classification was graded. We calculated the ROC AUC, sensitivity and specificity for thyromental height test as a primary end point of our study.

**Results:**

Only thyromental height test and Cormack-Lehane scale proved significant on occurrence of difficult intubation. The optimal sensitivity and specificity values of thyromental height test were met with a cut off value of 50 mm. With 1 mm increase in thyromental height test, risk of difficult intubation decreased by 7%.

**Conclusion:**

Thyromental height test is a simple, easy to perform and non-invasive test to predict difficult intubation in patients scheduled for elective double lumen tube intubation during thoracic surgical procedures. With 1 mm above 50 mm increase in thyromental height test the risk of difficult intubation decreased by 7%.

**Trial registration:**

Clinicaltrials.gov Identifier: NCT02988336.

## Introduction

Securing the airway and providing adequate oxygenation and ventilation is crucial in patients having surgery under general anesthesia. Difficulties during airway management may cause complications including hypoxemia, aspiration, airway trauma, and even death [1–4]. Difficulties during intubation, usually defined as a Cormack & Lehane Classification III and IV, are relatively common with a reported incidence ranging from 1 to 20% [5–11].

**Fig 1:**
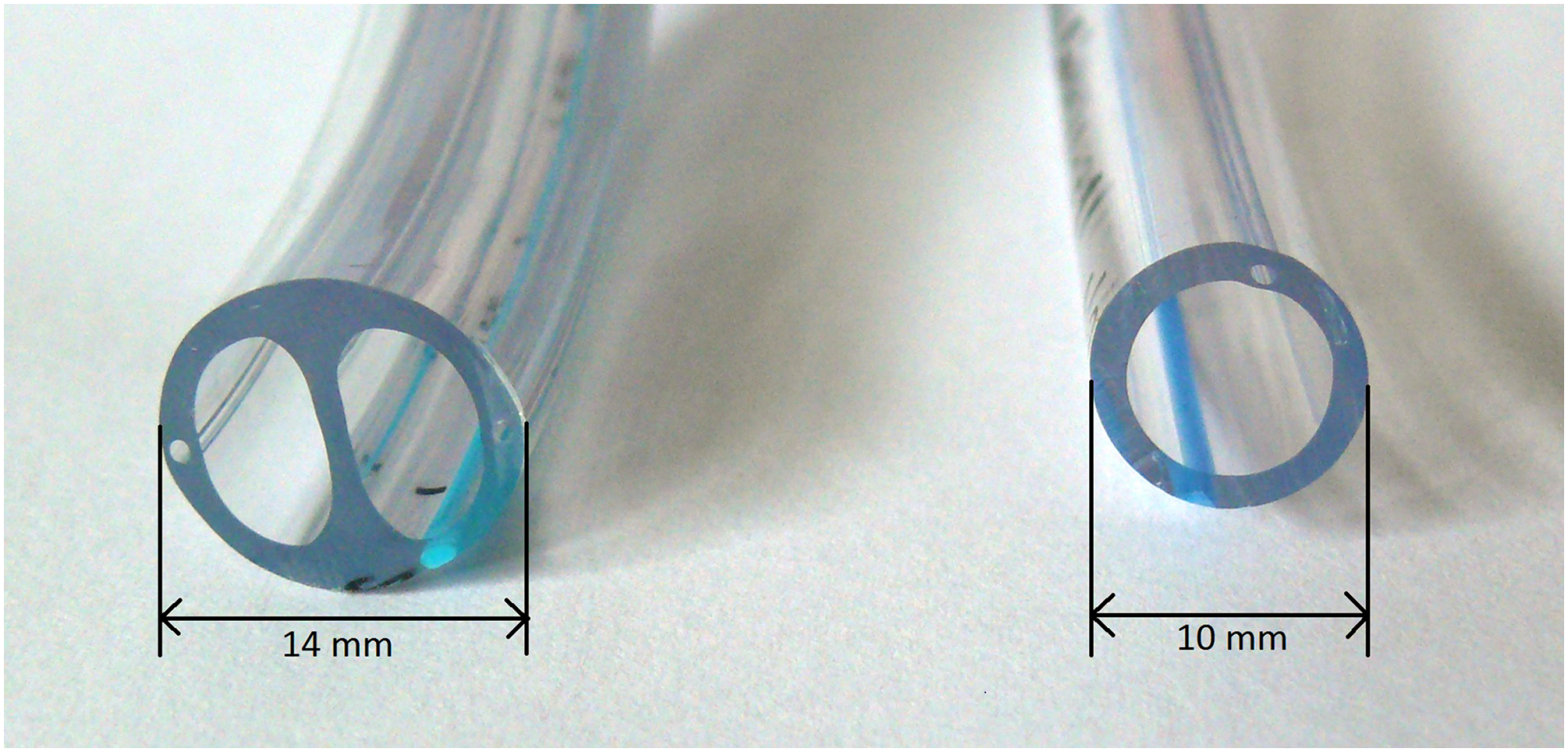
Diameter of a double lumen tube size: 37CH (left) and single lumen tube size: 8.0 I.D (right).

Single-lung ventilation is required for various surgical procedures, with thoracic surgeries being the most frequent indication. Double-lumen tubes (DLT) are the most commonly used devices in order to facilitate single-lung ventilation and collapse of the operated lung [11,12]. DLT’s are much larger (Fig 1), more rigid and bulkier than conventional single-lumen tubes and consequently are more likely to cause airway injuries and more difficult to place, despite good glottis visualization.

**Fig 2:**
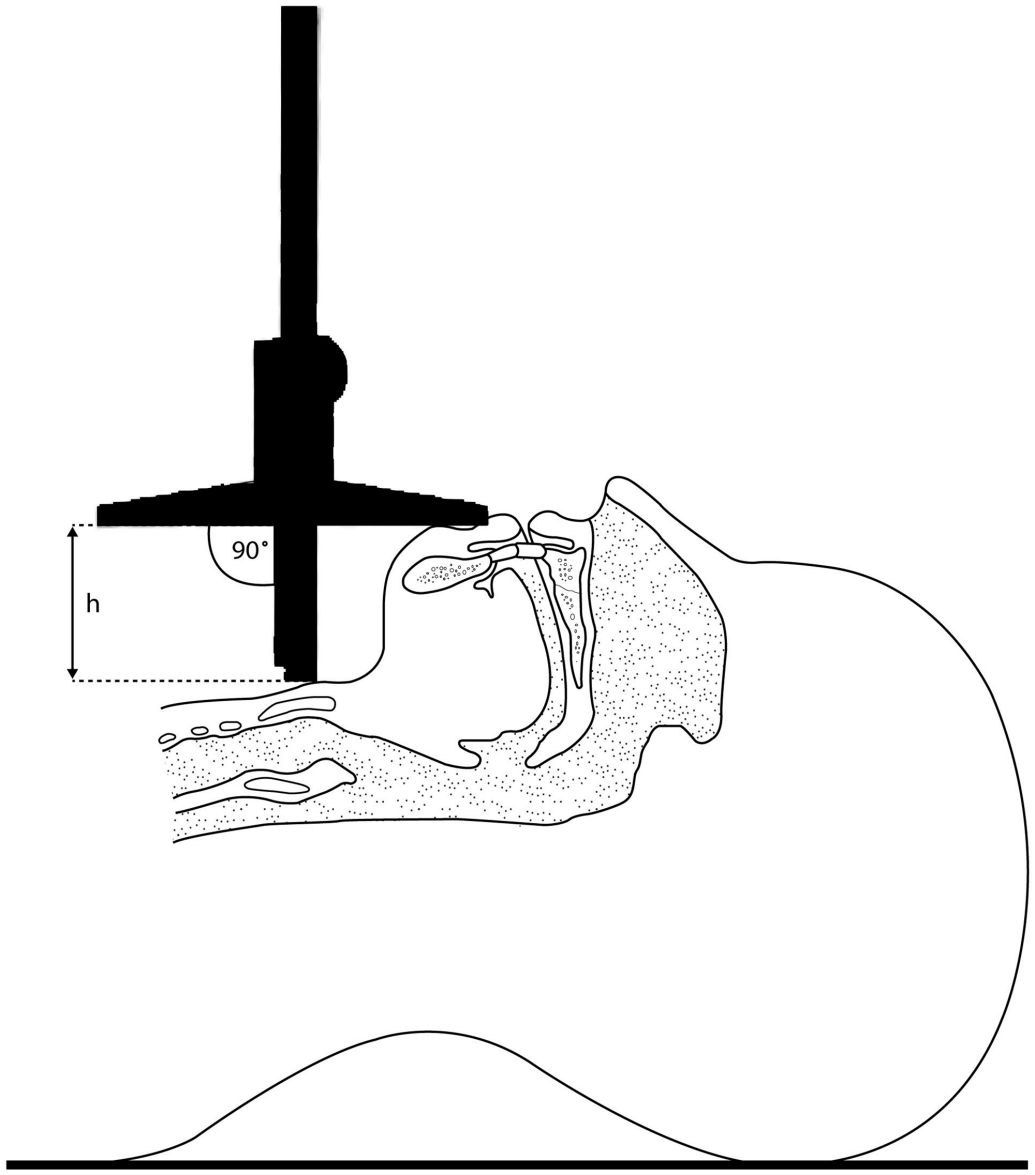
Measurement of thyromental height using a depth gauge.

Anesthesiologists usually try to limit the risk of unexpected difficulties and airway injuries during airway management. Therefore, several risk scores for difficult intubation have been proposed, but sensitivity is mostly limited and consequently, useful clinical guidance often fails [1,7,13–15]. The Thyromental Height test (TMHT) is an easy-to-do and non-invasive test. The test is based on the height between the anterior border of the mentum and the thyroid cartilage, while the patient lies supine with the mouth closed (Fig 2) [10]. Recent studies reported the TMHT to be more accurate to predict difficult intubation, compared to widely used purely anatomical measurements [10,16]. Clinical studies are lacking, therefore the goal of this study was to determine clinical performance and usefulness of the TMHT as a predictor of difficult intubation. In particular, we tested the hypothesis, that decreased TMHT is associated with higher risk of difficult intubation. Rate of difficult intubation served as our primary outcome.

## Material and methods

This study was approved by the Bioethics committee at Medical University of Silesia, Katowice, Poland. (Identifier: KNW/0022/KB1/43/I/16) on September 27, 2016. This study was also registered before starting enrollment at clincialtrials.gov (Identifier: NCT02988336).

**Fig 3:**
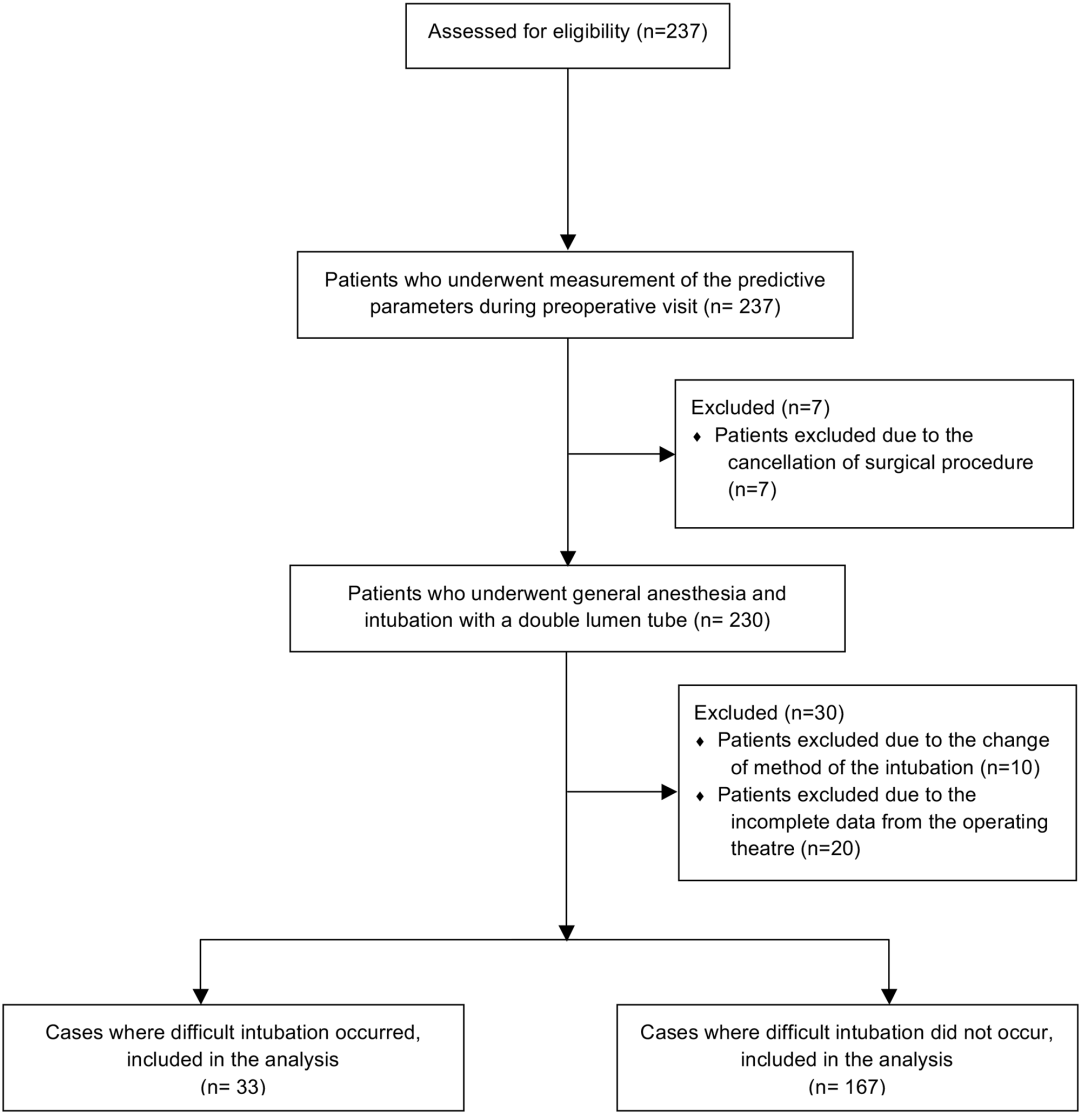
CONSORT flow chart.

After obtaining written consent, 237 adults having elective thoracic surgery requiring single-lung ventilation between October 2016 and January 2017 at the Department of Anesthesiology and Intensive Care, Medical University of Silesia, Poland were included in this study (Fig 3). Patients having emergency procedures, obvious anatomical abnormalities and indicated fiberoptic awake intubation were excluded. During obligate preoperative anesthetic visit, demographics were assessed and physical examination was performed by an anesthesiologists. A member of the study team, consisting of anesthesiologists and not involved in the perioperative part of this study, obtained the following predictive measurements:

- Modified Mallampati test (MMT): oropharyngeal view was assessed using the modified Mallampati classification. Patients were in sitting position with mouth maximally opened, tongue protruded and without phonation.
- Thyromental distance (TMD): measured between the thyroid prominence and the most anterior part of the mental prominence of the mandible with a tape measure (Standard, Hoechstmas, Sulzbach, Germany) as a distance in centimeters, with the patient in supine position, head fully extended, mouth closed.
- Sternomental distance (SMD): measured between the superior border of the manubrium sterni and the most anterior part of the mental prominence of the mandible with a tape measure (Standard, Hoechstmas, Sulzbach, Germany) as a distance in centimeters, with the patient in supine position, head fully extended, mouth closed.
- Thyromental height test (TMHT): measured as a height between the anterior border of the thyroid cartilage (on the thyroid notch just between the 2 thyroid laminae) and the anterior border of the mentum (on the mental protuberance of the mandible) with a depth gauge (21460605, Limit, Alingsås, Sweden) in millimeters, with the patient in supine position, head in neutral position and closed mouth.

The perioperative part of this study were performed by highly experienced staff anesthesiologists, each are having considerable experience in using DLT. All patients underwent general anesthesia according to a standardized protocol. Patients were premedicated according to their body weight, those up to 70 kg were given 7.5 mg midazolam orally, between 70 kg and 90 kg 10.5 mg midazolam orally, and those exceeding 90 kg 15 mg midazolam orally.

All patients were placed in the supine position on the operating table. Patients were preoxygenated, and general anesthesia was induced with propofol ~ 2 mg·kg^-1^, fentanyl ~ 2 μg·kg^-1^, and cis-atracurium ~ 0.15 mg·kg^-1^. Additional medication was given as necessary and complete muscle relaxation was confirmed by the absence of palpable twitches in response to supra-maximum train of four stimulation of the ulnar nerve at the wrist. Patients head was placed in optimal sniffing position. All intubation procedures were performed using a Macintosh blade in appropriate size, usually blade size 3 or 4 and a Robertshaw DLT in appropriate size, usually 37 for women and 39 for men.

Correct tube placement was confirmed by auscultation and capnography. If tube was misplaced or correct placement was impossible within 3 intubation attempts, the staff anesthesiologist deliberately decided to change the intubation device including use of videolaryngoscopy, Tube exchanger or combination of a single-lumen tube with a bronchial blocker [17]. Once intubation procedure was completed, general anesthesia was maintained according to local standard of care.

Best visualization of the airway was rated according to the Cormack & Lehane classification [18].

Failed intubation attempts were a prior defined as

- intubation attempt lasting more than 3 minutes, or
- tube misplacement into esophagus,
- removal of the laryngoscope from the oral cavity and intermittent ventilation using an anesthesia bag

Difficult intubation was a priori defined as:

- more than two attempts needed to achieve successful intubation with a conventional laryngoscope with a Macintosh blade, or
- two failed intubation attempts undertaken by two experienced anesthesiologists, or
- a change in technique was necessary (ex. different upper airway management device, different type of laryngoscope, bougie),

### Statistical analysis

Statistical analysis was performed using STATISTICA 10.0 (StatSoft, Cracow, Poland). Variance analysis and T- Student test for variables with distributions similar to normal were used. Otherwise, Kruskal-Wallis and U Mann–Whitney test were performed. Descriptive variables were compared using Pearson’s χ^2^ test. To evaluate the impact of potentially influential factors on the occurrence of difficult intubation, a logistic regression analysis was performed. The area under the receiver operating characteristic (ROC) curve for the TMHT was used to show the predictive values of various thyromental height distances. The area under the ROC curve and sensitivity and specificity graph were used to calculate the ideal cut off point for TMHT. The type I error was set to a = 0.05. To account for multiple testing, criticalwas adjusted using the Bonferroni method (a = 0.0125). To avoid collinearity in our regression we compute the variance inflation factor and look for variables with a high variance inflation factor. If the variables were ordinally scaled, we computed Spearmon’s rank correlation coefficients for several pairs of variables and compare the computed value with a certain threshold. For variables nominally scaled we perform a pairwise chi-square test for independence. We have transformed all categorical variables into dummies in order to have reference groups and interpret odds-ratios.

Based on previous studies we calculated the necessary sample size with at least 160 participants using G*Power 3.1 (two-tailed t-test; Cphen’s d0.8, alpha error:0.05, power:0.95) [19,20].

The results are given as median with odds ratio (OR—odds ratio) and 95% confidence intervals. Results were considered statistically significant with p <0.05.

## Results

237 patients were enrolled in this study. Overall 37 patients had to be excluded from analysis: surgery was cancelled in 7 patients, missing data from 20 patients, and change of intubation device (single-lumen tube instead of a DLT) in 10 patients. Finally, the remaining 200 patients (76 female and 124 men) completed the study and were included in our statistical analysis. Demographics are presented in Table 1.

**Table 1:** Demographic data of the patients and comparison of studied predictive factors depending on occurrence of difficult intubation. Values are mean, standard deviation (SD) or number.

	Difficult intubation (n = 33)	Normal intubation (n = 167)	p-value
Age (years)	60 ± 8.4	61 ± 12	0.476
Height (cm)	174 ± 8	174 ± 7	0.147
Weight (kg)	95 ± 22.3	79.6 ±15	**0.004**
Body Mass Index (kg/m^2^)	31 ± 6	26 ± 5	**0.006**
Thyromental height; mm ± SD	46 ± 10	54 ± 9	**0.001**
Thyromental distance; mm ± SD	93 ± 21	95 ± 18	0.37
Sternomental distance; mm ± SD	172 ± 33	179 ± 23	0.85
Mallampati scale;			0.24
I	16	94	
II	7	52	
III	6	14	
IV	4	7	
Cormack & Lehane classification;			
I	12	112	**<0.001**
II	7	43	
III	10	11	
IV	4	1	

Logistic regression analysis revealed that only TMHT and Cormack-Lehane scale have significant impact on the occurrence of difficult intubation (Table 3, Fig 3). With every 1 mm increase in THMT, the risk of difficult intubation decreased by 7%. While the increase in Cormack—Lehane scale in 1 point is associated with a 3-fold increased risk of difficult intubation. Other factors, namely the thyromental distance, sternomental distance and Mallampati score turned out to be irrelevant to the occurrence of difficult intubation in developed statistical model.

77 (38.5%) of patients underwent thoracotomy and 123 (61.5%) underwent a video assisted thoracoscopy (VATS). Difficult intubation occurred in 33 of all patients, representing an overall incidence of 16.5%.

Patients with difficult intubation had a significant lower thyromental height (46 mm vs 54 mm, p = 0.001) and lower Cormack & Lehane classification (p = 0.0001; Table 1). Thyromental distance, Sternomental distance and Mallampati score did not differed significantly between the normal and difficult intubation groups (Table 1).

Odds ratios were calculated for the predictive factors. Results are indicating that increase of thyromental height and increase of the Cormack & Lehane classification is statistically significant (Table 2).

**Table 2:** Impact of studied factors on difficult intubation. Values are odds ratio (OR), 95% confidence intervals (95% CI).

	OR	95% CI	p
Increase in thyromental height test	0.93	0.88–0.98	0.007
Increase in Cormack & Lehane classification	2.97	1.69–5.24	0.0001
Decrease in thyromental distance	1.46	0.95–2.26	0.086
Decrease in sternomental distance	0.88	0.68–1.13	0.31
Increase in Mallampati scale	1.3	0.77–2.18	0.32

**Fig 4:**
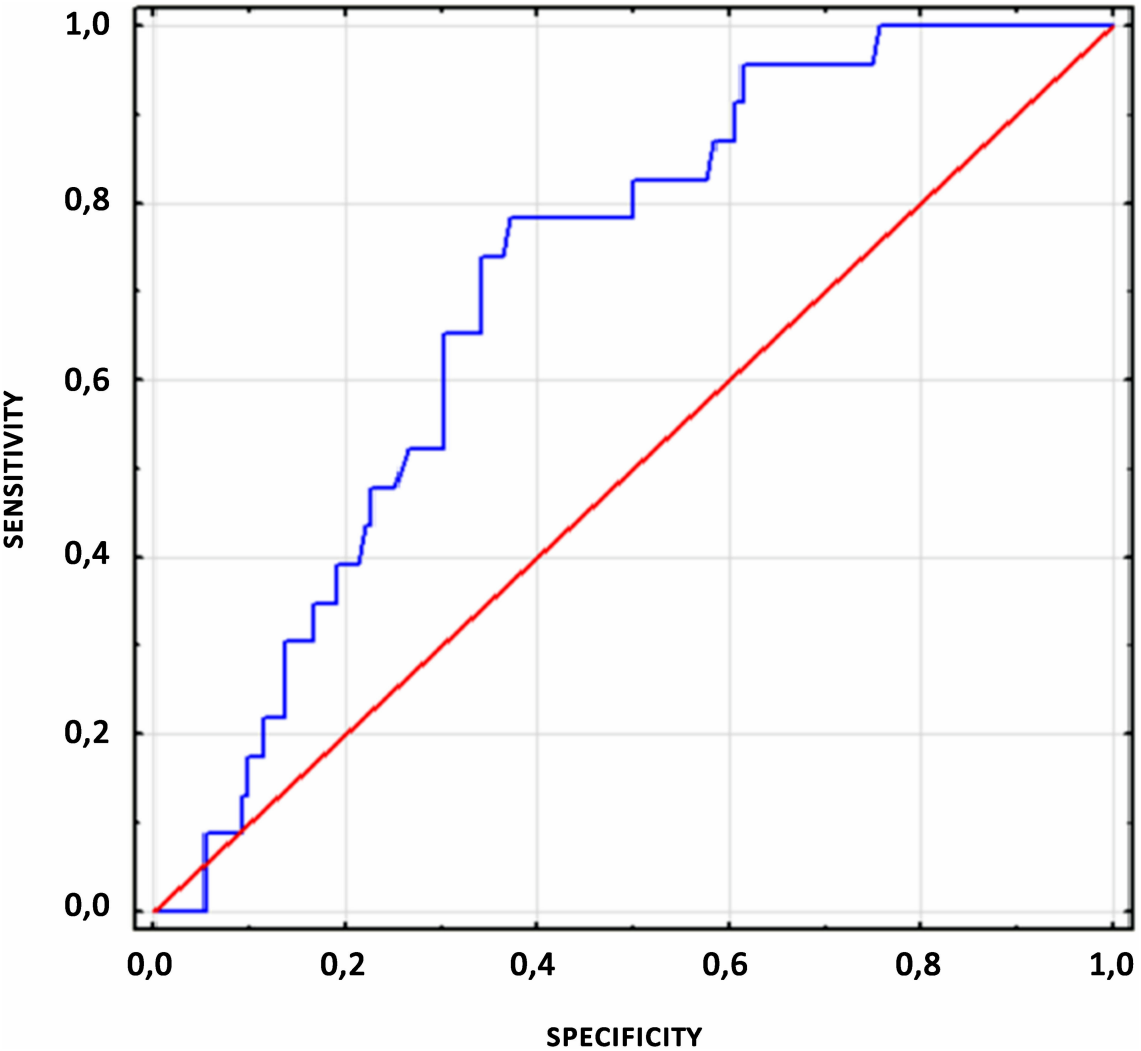
ROC curve for thyromental height test.

**Fig 5:**
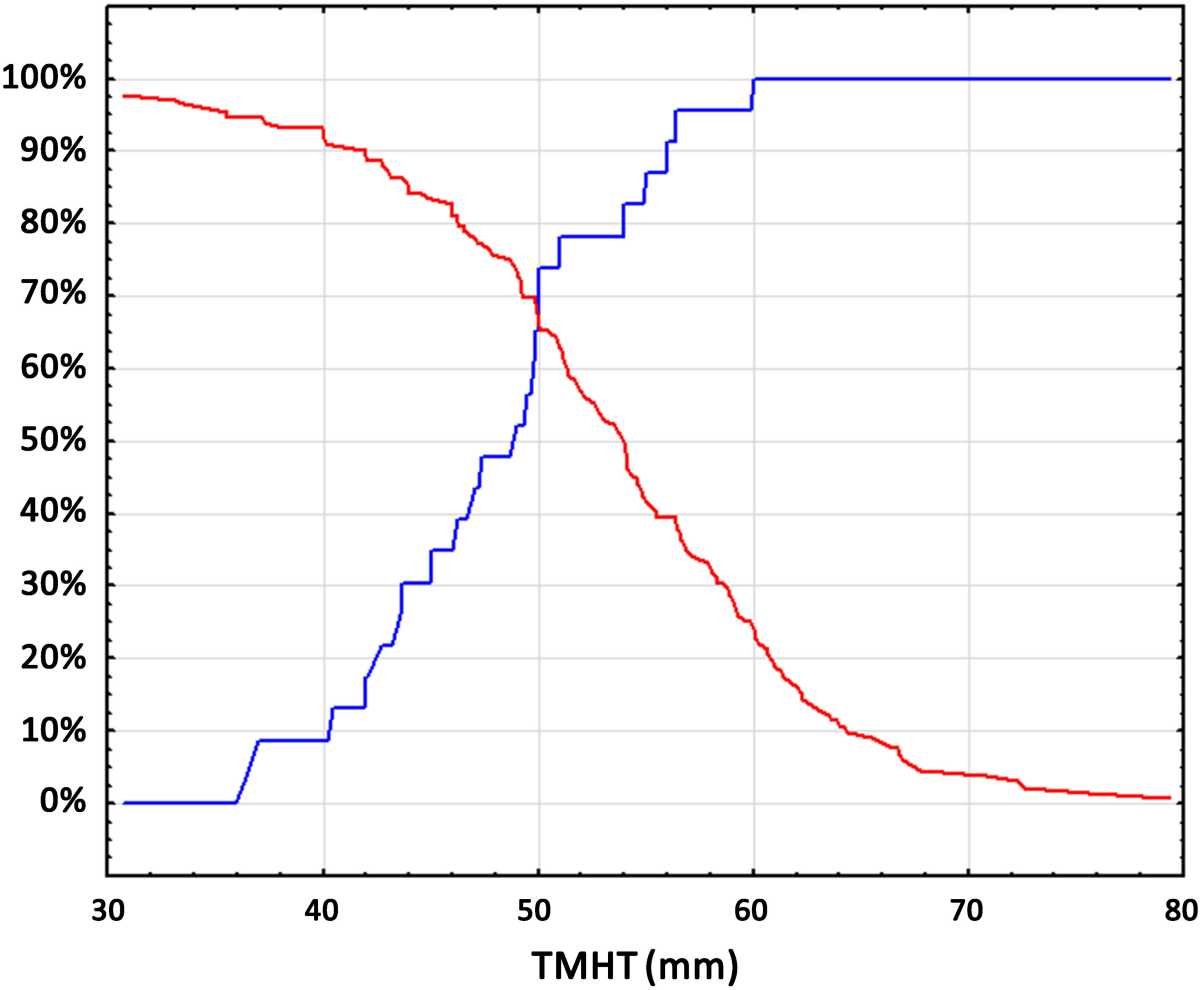
Sensitivity and specificity graph for thyromental height test with a 50 mm cut-off value.

Among the preoperative factors that could affect the occurrence of difficult intubation only thyromental height proved to be significant. The ROC curve (Fig 4) and graph sensitivity—specificity (Fig 5) were plotted for thyromental height. The optimal cut off value for TMHT was 50 mm, with a 70% sensitivity and 70% specificity. The NPV value with the TMHT value> 51 cm is 85%. Sensitivity and specificity for other scales used in this trial are presented in Table 3.

**Table 3:** Sensitivity and specificity parameters.

Test	AUC	Sensivity	Specificy	PPV	NPV
TMHT	0.573	70%	70%	17%	85%
Thyromental distance	0.623	50%	36%	24%	93%
Sternomental distance	0.535	60%	41%	19%	87%
Circuit neck	0.707	67%	79%	33%	88%
Cormack-Lehane grade	0.801	65%	92%	62%	93%
Mallampati grade	0.563	25%	87%	26%	86%

**Fig 6:**
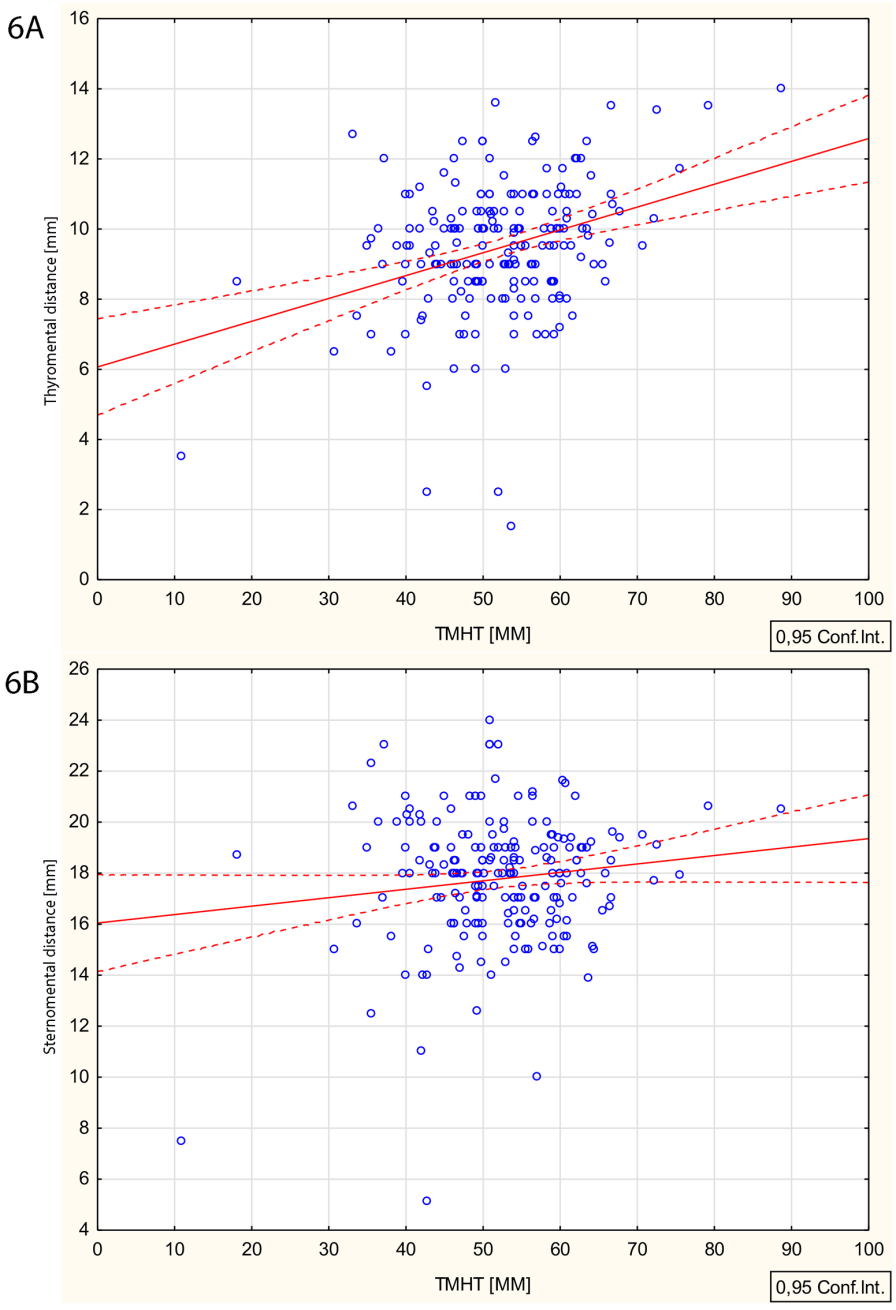
Simple linear regression analysis between (6A) TMHT height and thyromental height and (6B) between TMHT height and sternomental height.

Simple linear regression analysis between TMHT height and thyromental height (R = 0.334, P<0.001) and between TMHT height and sternomental height (R = 0.129, P = 0.070) are presented in Fig 6.

## Discussion

Prediction of difficult intubation is of high clinical interests and several anatomical landmarks and multifactorial indexes have been developed in the past [5–7,10,13,15,21]. Multifactorial indexes are more reliable than single measures, and no anatomical landmark alone has been reported to have acceptable accuracy for prediction of difficult intubation so far [10]. Identifying a single easy-to-do and repeatable predictor of difficult intubation is still lacking.

The TMHT is an already long known technique, but recently gained popularity again. Etezadi et al. investigated the predictive capacity compared to other single measures including the modified Mallampati test, thyromental distance and the sternomental distance [10]. The sensitivity of the TMHT was reported to be approximately 83% (CI, 74–88%), and the specific value of the TMHT approximately 99.3% (CI 96%–99.98%). The TMHT is an inexpensive, and easy to perform measure. Consequently the TMHT might be an appropriate predictive sole measure. Our study confirms the findings by Etezadi et al, as the TMHT was the superior single measure for predicting difficult intubation, although Etezadi et al. performed intubation in patients requiring single-lumen tubes, instead of using DLT in our group.

The thyreomental distance is an easy-to-measure anatomical measurement, and is commonly used in the clinical setting. However, the measurement is a surrogate for inadequate head extension, rather than dimension of the submandibular space [22]. Results of our study indicate, that decrease of the thyreomental distance did not predict difficult intubation.

The sternomental distance is commonly used in clinical practice, although repeatedly reported to be a non-adequate sole predictor of difficult intubation [23]. Contrary, a combination of several sole measures including the sternomental distance appears to be more reliable [23,24].

The Mallampati score is a widely used airway measurements, although there is strong evidence, that the Mallampati score does not predict difficult intubation. Our results confirm these previous findings, as the Mallampati Score was not predictive.

The Cormack & Lehane classification was already introduced into clinical practice several decades ago and represents the most commonly used predictor of difficult intubation [18]. However, assessment of the Cormack & Lehane classification requires laryngoscopy, an invasive procedure and therefore, is not suitable in the preoperative assessment of potential difficult intubation.

The incidence of difficult intubation was 16.5% in our study, which perfectly fits within the previously reported range between 1 and 20% [5–11].

The findings of our study are limited by several limitations: First, our study was limited to patients scheduled for elective surgery and having no history of significant difficult intubation (indicated fiberoptic awake intubation). Thus result of this study are applicable to these patients. Second, we investigated single measures only, but combination of multiple measures might further increase sensitivity. Third, the TMHT is usually measured using a semi-electronic device and there is a potential for any learning effect, andcertain inter-observers variability, especialy if a depth gauge is not used. Fourth, examinations were performed by the study team, highly familiar with all test performed. Clinical practice might differ, especially if rarely used measurements would have been used or less experienced providers would have performed the measurements. Last, this study intended to proof, that the TMHT is a reliable test, but results of this study has be proven by other major studies.

## Conclusions

Thyromental height test is a simple, easy to perform and non-invasive test to predict difficult intubation in patients scheduled for elective double lumen tube intubation during thoracic surgical procedures. With 1 mm above 50 mm increase in thyromental height test the risk of difficult intubation decreased by 7%.

## Supporting information

S1 Table New method of difficult intubation prediction. Study protocol.
(DOCX)

S1 Checklist Consolidated standards of reporting trials checklist. (DOC)
