# Supplementary material for: Thyromental height test as a new method for prediction of difficult intubation with double lumen tube
Source: PLoS One. 2018 Sep 13;13(9):e0201944. doi: 10.1371/journal.pone.0201944 (PMC6136707; doi:10.1371/journal.pone.0201944)
Supplement: S1 Table — Study protocol. (DOCX) [file pone.0201944.s001.docx]

TMHT - New Method of Difficult Intubation Prediction

Study protocol

Proper prediction of the occurrence of difficult intubation plays a crucial role in anaesthesiology. For years attempts have been made to develop methods allowing anaesthesiologists to correctly predict the occurrence of difficult intubation. None of the currently used tests and anthropometric measurements present satisfying predictive value. Recent studies show promise in Thyromental Height Test (TMHT), which base on the height between the anterior borders of the mentum and the thyroid cartilage, measured while the patient lies in the supine position with closed mouth, as a single, non-invasive predictor of difficult intubation. Use of double lumen endotracheal tubes during thoracic procedures, due to greater diameter of the tube, may lead to higher occurrence of difficult intubation. That is why, the purpose of this study is to assess the usefulness of the TMHT in prediction of difficult intubation using double lumen endotracheal tubes in patients scheduled for elective thoracic procedures and relate it to other, commonly used predictive tests.

During routine, preoperative anaesthetic visit thyromental height, thyromental distance, sternomental distance and Mallampati scale score are assessed. Then, during direct laryngoscopy and intubation, score in Cormack-Lehane scale and occurrence of difficult intubation are noted.

**Purpose**

The purpose of this study is to assess the usefulness of the Thyromental Height Test in prediction of difficult intubation using double lumen endotracheal tubes in patients scheduled for elective thoracic procedures

**Primary Outcome Measures:**

**Thyromental height.** The height between the anterior border of the thyroid cartilage (on the thyroid notch just between the 2 thyroid laminae) and the anterior border of the mentum (on the mental protuberance of the mandible), in supine position with mouth closed, measured with a depth gauge, measured during routine preoperative anaesthetic visit.

**Secondary Outcome Measures:**

**Thyromental distance.** The distance between the thyroid prominence and the most anterior part of the mental prominence of the mandible, measured with a standard centigrade ruler as the distance in centimetres with the patient in supine position, head fully extended, mouth closed, during routine preoperative anaesthetic visit.

**Sternomental distance**. The distance in centimetres between the superior border of the manubrium sterni and the bony point of the mentum, with the patient in supine position, head fully extended, mouth closed, measured with a standard centigrade ruler, during routine preoperative anaesthetic visit.

**Score in modified Mallampati test**. The oropharyngeal view is assessed in sitting position, mouth maximally opened, tongue protruded, without phonation, measured during routine preoperative anaesthetic visit.

**Score in Cormack-Lehane scale**. During direct laryngoscopy the patient is graded in Cormack-Lehane Scale by the laryngoscopist.

**Study Population**

Consecutive patients undergoing elective thoracic procedures in university hospital, requiring general anaesthesia, elective direct laryngoscopy and intubation with a double lumen endotracheal tubes.

Inclusion criteria: patients scheduled for elective thoracic procedures, requiring general anaesthesia, direct laryngoscopy and intubation with a Robertshaw type double lumen endotracheal tubes, consent for participation in the trail, older than 18.

Exclusion Criteria: emergency procedures, visible anatomic abnormalities, patients scheduled for awake fibre optic intubation, lack of consent for participation in the trail.

**Research card**

| **Name and Surname** | |  |  |
| --- | --- | --- | --- |
|  | |  |  |
| **Age** |  | **TMHT** |  |
| **Gender** |  | **Thyromental distance** |  |
| **Body weight** |  | **Sterno-mental distance** |  |
| **Height** |  | **Cormacka-Lehane score** |  |
| **BMI** |  | **Mallampi score** |  |
| **Toothing** | Full | **Carlens tube size** |  |
|  | Partial upper | **type of surgery** |  |
|  | Partial down | **Difficult intubation** | Yes / No |
|  | Lack |  |  |
| **Neck circumference** |  | **Three finger test** |  |
